# Geographical shifts in the successional dynamics of inland dune shrub communities

**DOI:** 10.1002/ece3.9828

**Published:** 2023-02-16

**Authors:** Sergio Chozas, Rosa M. Chefaoui, Otília Correia, Ana M. C. Santos, Joaquín Hortal

**Affiliations:** ^1^ cE3c – Centre for Ecology, Evolution and Environmental Changes and cE3c – Centre for Ecology, Evolution and Environmental Changes & CHANGE – Global Change and Sustainability Institute, Faculdade de Ciências Universidade de Lisboa Lisbon Portugal; ^2^ Área de Biodiversidad y Conservación Universidad Rey Juan Carlos Móstoles Spain; ^3^ Terrestrial Ecology Group (TEG‐UAM), Departamento de Ecología, Facultad de Ciencias Universidad Autónoma de Madrid Madrid Spain; ^4^ Centro de Investigación en Biodiversidad y Cambio Global (CIBC‐UAM) Universidad Autónoma de Madrid Madrid Spain; ^5^ Department of Biogeography and Global Change, Museo Nacional de Ciencias Naturales (MNCN‐CSIC) Madrid Spain

**Keywords:** biogeography, community dynamics, disturbance history, habitat suitability, scenopoetic variables, soil organic matter, *Stauracanthus genistoides*, *Ulex australis*

## Abstract

Species' environmental requirements and large‐scale spatial and evolutionary processes determine the structure and composition of local communities. However, ecological interactions also have major effects on community assembly at landscape and local scales. We evaluate whether two xerophytic shrub communities occurring in SW Portugal follow constrained ecological assembly dynamics throughout large geographical extents, or their composition is rather driven by species’ individualistic responses to environmental and macroecological constraints. Inland dune xerophytic shrub communities were characterized in 95 plots. Then, we described the main gradients of vegetation composition and assessed the relevance of biotic interactions. We also characterized the habitat suitability of the dominant species, *Stauracanthus genistoides*, and *Ulex australis*, to map the potential distribution of the xerophytic shrub communities. Finally, we examined the relationships between the vegetation gradients and a broad set of explanatory variables to identify the relative importance of each factor driving changes in community composition. We found that xerophytic shrubs follow uniform successional patterns throughout the whole geographical area studied, but each community responds differently to the main environmental gradients in each region. Soil organic matter is the main determinant of community variations in the northern region, Setúbal Peninsula, whereas aridity is so in the South/South‐Western region. In contrast, in the central region, Comporta, the variation between *S. genistoides* and *U. australis* communities is explained mainly by aridity and temperature seasonality, followed by the individualistic responses of the dominant species and soil organic matter. Overall, these results indicate that, the relative importance of the main factors causing community‐level responses varies according to regional processes and the suitability of the environmental conditions for the dominant species in these communities. These responses are also determined by intrinsic community mechanisms that result in a high degree of similarity in the gradient‐driven community stages in different regions.

## Graphical abstract

In this work, we assess the role of both individualistic species responses to the environment and local processes in the composition and structure of xerophytic scrub communities in the SW Iberian Peninsula by evaluating the geographical consistency of the variations along the compositional gradients between communities dominated by *Stauracanthus genistoides* and those dominated by *Ulex australis* throughout most of the area of co‐occurrence of both species. We found that xerophytic shrubs follow uniform successional patterns throughout the whole geographical area, but also that the relative importance of the main factors causing community‐level responses varies according to regional processes and the suitability of the environmental conditions for the dominant species in these communities.

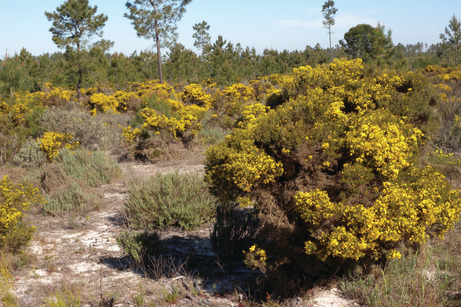

## 1 INTRODUCTION

The origin and nature of ecological communities is a central question in ecology (Mittelbach & Schemske, 2015). A significant body of evidence points to large‐scale evolutionary and biogeographical processes as major determinants of the composition and structure of local communities, through both individualistic responses of species to environmental gradients and historical effects (Hawkins, 2008; Lessard et al., 2012). This led to Ricklefs' (2008) claim for the ‘disintegration’ of ecological communities, where the outcome of ecological interactions would be selected along long‐term coevolutionary processes operating at the regional scale (Ricklefs, 2011, 2015). Despite such emphasis on large‐scale processes, community dynamics are unavoidably mediated by local interactions (e.g., Ings et al., 2009; Kraft et al., 2014), which determine the ecological role of local populations (Polis et al., 2004), and ultimately affect their spatial distributions (Soberón, 2007). Therefore, species distributions are shaped by both environmental requirements and the interactions with other species (HilleRisLambers et al., 2012; Wisz et al., 2013), which are intrinsically mediated by the dispersal potential of each species. These interactions follow a scaling trend toward more deterministic importance at local and landscape scales than at larger scales (Araújo & Rozenfeld, 2013; Hortal et al., 2010). Consequently, the species present locally are determined by processes acting at different scales, which ultimately affect the diversity and structure of communities (Medina et al., 2018; Pärtel et al., 2016).

This local vs regional dichotomy poses a dilemma about how ecological communities are formed (Cornell & Harrison, 2014). From the top‐down perspective typically adopted in biogeography and macroecology, community composition would be the result of macroecological constraints acting on a species pool with analogous environmental requirements (D'Amen et al., 2017; Guisan & Rahbek, 2011) and filtered by ecological assembly rules (i.e., dispersal, abiotic and biotic restrictions on community structure and composition sensu Götzenberger et al., 2012). According to this view, a series of ecological filters unfold hierarchically, where each filter reduces the number (and identity) of the species that occur at each scale, from the global pool of species down to local ecological communities (D'Amen et al., 2017; Guisan & Rahbek, 2011). This implies that species are first filtered by large‐scale environmental gradients, following a characteristic and well‐defined set of requirements (Gleason, 1926; Hortal, De Marco Jr, et al., 2012; Ricklefs, 2008). A simplistic view of this assumption is that species co‐occurrence mainly determines their local population dynamics, but not their responses to the environment. This contrasts with the widespread evidence that interactions can affect species’ responses to biotic and abiotic gradients (see, e.g., Bulleri et al., 2016; Crotty & Bertness, 2015; Facon et al., 2021; Godsoe et al., 2017; Johnson, 1975). These interactions can shift the prevalence of equalizing versus stabilizing forces (Bartomeus & Godoy, 2018), hence changing species' realized niche along several dimensions (Colwell & Rangel, 2009; Hutchinson, 1978). Such changes can lead to different responses along successional gradients (Backhaus et al., 2021), thereby making it difficult to distinguish between the effects of environmental filters and biotic interactions (Kraft et al., 2014).

**FIGURE 1:**
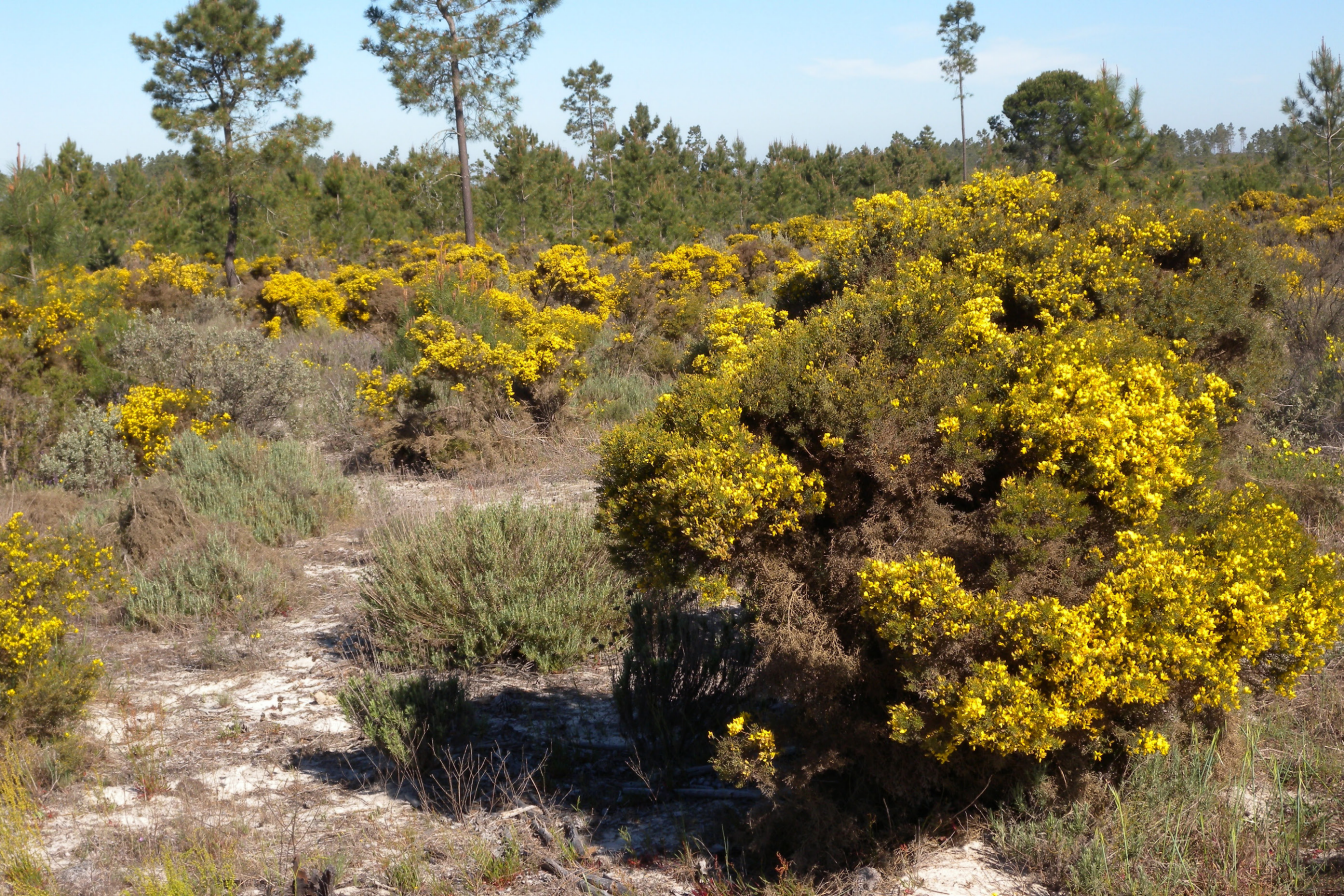
Inland dune shrub community dominated by *Stauracanthus genistoides* occurring on sandy soils in Comporta, Portugal.

Here we analyze a comprehensive survey of semiarid shrub communities growing on inland dunes to assess the relative importance of local and mesoscale gradients, and the individualistic responses of species, on successional changes in community composition. Dominant shrub communities growing on inland dune habitats of the Setúbal Peninsula‐Alentejo littoral region (Portugal) follow a clear compositional gradient, from *Stauracanthus genistoides* to *Ulex australis*‐dominated scrubs (Chozas, Correia, et al., 2015; Neto, 2002) (Figure 1). In this relatively small region, both communities represent the extremes of a Mediterranean xerophytic shrub series driven by soil organic matter (Chozas, 2017; Chozas, Correia, et al., 2015). Therefore, in this work, we assess whether this local factor is consistently the main driver of the dynamics of these communities along their whole geographic gradient in SW Portugal, or alternatively, other additional factors acting at different scales also determine their dynamics. To do this, we first evaluate whether the changes between *S. genistoides* and *U. australis‐*dominated communities occur consistently throughout the wider extent of all inland dune habitats of the region where both species coexist. Then, we assess whether variations in community composition can be explained by factors affecting xerophytic shrub communities at different scales: (a) soil organic matter (SOM) measured locally; (b) mesoscale climatic gradients such as aridity (Chozas, Correia, et al., 2017); and (c) the individualistic responses to gradients of soil type and climate of *S. genistoides* and *U. australis*, measured as the habitat suitability for each species at a large scale. If local processes are preponderant, we expect that the importance of soil organic matter will remain similar throughout the whole studied region. In contrast, if communities are mainly responding to geographical variations along the study regions, then the importance of mesoscale environmental gradients and habitat suitability will increase with the extent of analysis. Finally, this study will help determine the relative importance of local and regional drivers in community dynamics at different spatial scales.

## 2 MATERIALS AND METHODS

### 2.1 Study area

**FIGURE 2:**
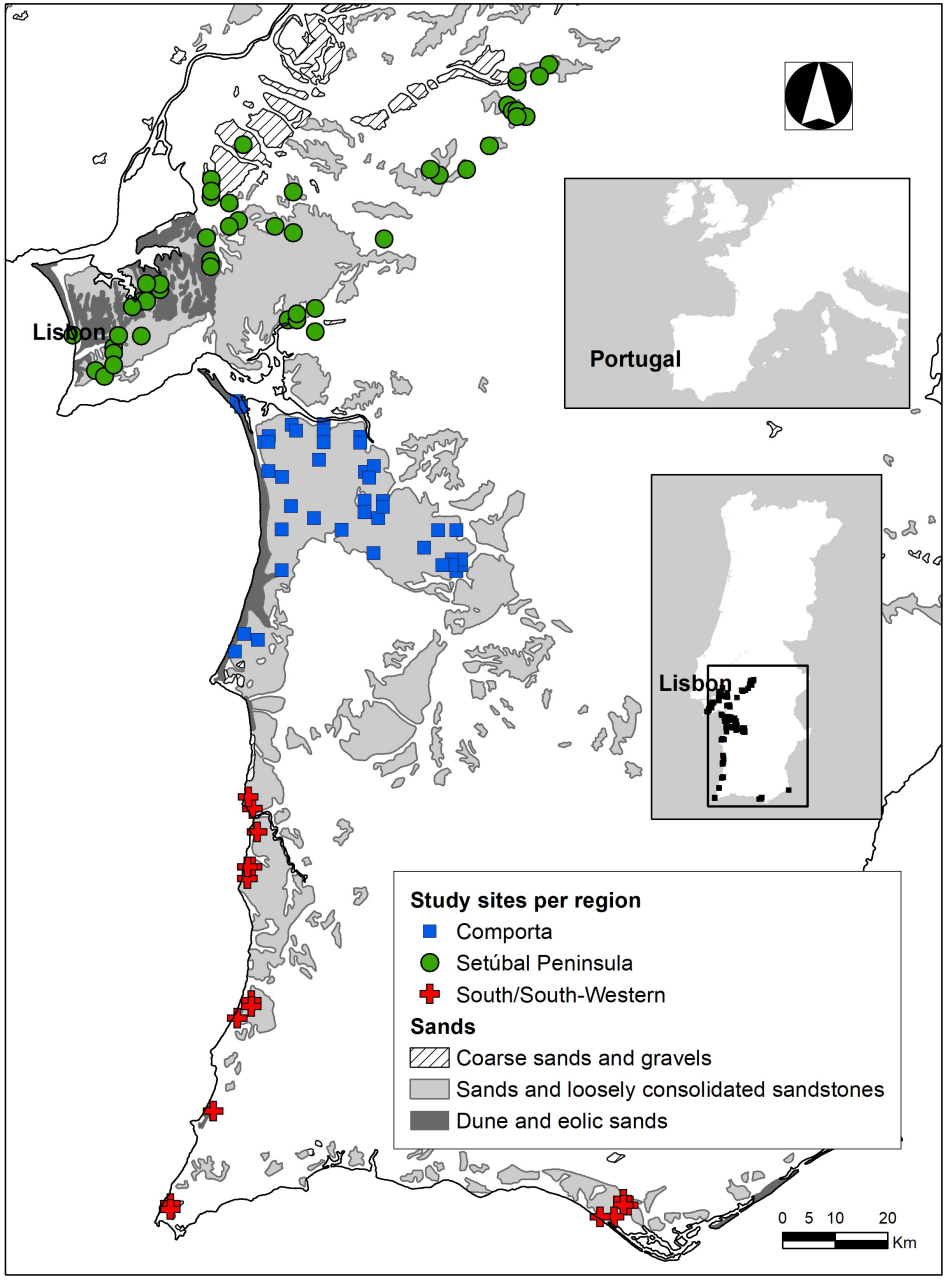
Study sites (colored symbols) and geographic distribution of the sandy soil formations in South‐West Portugal (areas in the map marked in gray and with diagonal lines). These formations constitute the potential habitat for the studied communities. Sites were divided into three regions—Comporta (blue squares), Setúbal Peninsula (green circles), and South/South‐Western region (SSW) (red triangles).

The study area included all inland sandy soils of South West Portugal, totaling 2900 km^2^ between the left margin of Tagus River (38°53′ N, 8°49′ W) and the Guadiana River Estuary (37°10 N, 7°24′ W) (Figure 2). Elevation varies from 0 to 152 m a.s.l. Climate is Mediterranean but with considerable intra and inter‐annual variations in mean monthly precipitation and mean monthly temperatures (SNIRH, 2019). The dominant soil type is podzol, with some regosols and cambisols (with moderate and very weak soil development, respectively) (APA, 1992). Vegetation is dominated by semi‐natural Maritime pine (*Pinus pinaster*) forests of variable density, ranging from open to dense formations with a xerophytic shrub community understory (Neto et al., 2004). The xerophytic shrub communities are dominated by two xerophilous thorny shrubs in the family Fabaceae: *Stauracanthus genistoides*—dominating the Thymo capitellati‐Stauracanthetum genistoides phytosociological association in sandy soils on stabilized or mobile dunes and sandy alluvial deposits, and *Ulex australis*—dominating the association Erico umbellatae‐Ulicetum welwitschiani on stabilized dunes and soils derived from acid sandstones (Chozas, Correia, et al., 2015; Neto et al., 2009). For simplicity herein we use *S. genistoides* or *U. australis* communities to refer to these formations.

### 2.2 Field surveys

Ninety‐five plant communities were sampled within the area of occurrence in Portugal of sandy xerophytic shrub communities (Figure 2). Survey sites were selected using the following procedures. First, we analyzed the distribution maps of *S. genistoides* and *U. australis* available in Flora‐on portal (https://flora‐on.pt/)—this portal, created by the Botanical Society of Portugal, contains occurrence data of vascular plants from the Portuguese flora collected by project collaborators (over 625,000 records as of January 2023)—to determine the area of occurrence of both communities. Then, in ArcGIS 10, we used two layers depicting: (i) sandy soils derived from the lithological map of Portugal (APA, 1992) and (ii) areas with forest and semi‐natural land use cover (Caetano et al., 2009). Finally, survey localities, herein called sites, were randomly distributed from these previously identified areas using Hawth's Analysis Tools for ArcGIS (Beyer, 2004). Sites were divided into three regions (Figure 2)—Setúbal Peninsula (42 sites), Comporta (33 sites), and South/South‐West (SSW herein, 20 sites), defined according to their geographical distribution, sand morphogenesis, and age (Figure 2), and dominant land uses at regional level (see Figure S1—Appendix S1). The Setúbal Peninsula is an industrialized and densely populated region that maintains scattered areas of well‐preserved shrublands, coniferous forests, and cork oak savannah‐like *montados*. The sites in this region were distributed on a heterogeneous matrix constituted by Pliocene littoral (i.e., coastal, fluvial, and deltaic) sands with some areas of loosely consolidated sandstones and Pleistocene coarse sands (Pais et al., 2012). Comporta sites were positioned on a Pleistocene continuous eolian‐sand formation with some Mio‐Pliocene outcrops of detrital deposits and dominated by semi‐natural coniferous forests. SSW sites were distributed along the Portuguese Southern coast on spatially restricted littoral sand formations with heterogeneous morphogenesis and highly diverse natural and artificial environments. Since no obvious physical barriers prevent dispersal between the three regions, we considered that they could be potentially colonized by the same pool of species (i.e., the filtered species pool sensu Zobel, 2016).

One 10 × 10 m quadrat was randomly placed at each study site. In the quadrat, the projected cover of shrub species (chamaephytes, microphanerophytes, and nanophanerophytes) was assessed using the line intercept method along four 10 m straight lines set at 2 m intervals. A composite soil sample for each site was collected by bulking five sample soils from the top 20 cm, four obtained at the corners of the 10 × 10 m quadrat and one in the center (Chozas, Correia, et al., 2015). Soil organic matter content was directly measured by dry combustion (adapted from ISO 10694:1995) from the soil samples; this procedure was done in the LAS/INIAV laboratory (Laboratório de Analises de Solos do Instituto Nacional de Investigação Agrária e Veterinária, Lisbon).

Plant nomenclature follows the checklist for the Portuguese flora (ALFA, 2010). Species were identified using *Flora Iberica* (Castroviejo, 1986–2022) and *Nova Flora de Portugal* (Franco, 1971–1984; Franco & Afonso, 1994–2003). Individual plants in several study sites presented intermediate characters between *Stauracanthus genistoides* and *S. spectabilis*. Taking this and the existence of some taxonomic discrepancies into account (Chozas, Chefaoui, et al., 2017; Pardo et al., 2008), as well as the similar ecology of both species, both taxa were considered as *S. genistoides* aggr. for our analyses. The two subspecies of *Ulex australis* (*U. australis* subsp. *australis and U. australis* subsp. *welwitschianus*) were also considered as a single taxon: *Ulex australis* aggr.

### 2.3 Statistical analyses

**FIGURE 3:**
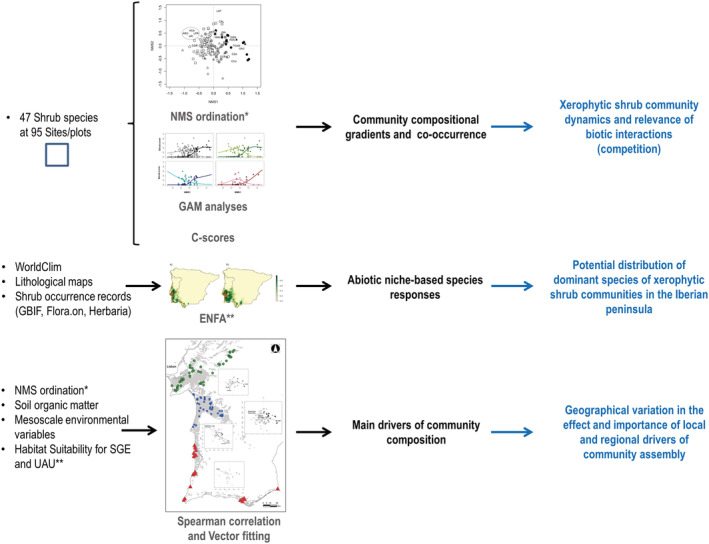
Schematic representation of the sequence of analyses (text in gray) conducted in this study, showing the data used (text in black), the phenomena studied (text in black bold), and the corresponding interpretation of each analysis (text in blue bold).

We assessed the geographic variations in species composition and related them with the abiotic responses to the environment of the two dominant species (*Stauracanthus genistoides* and *Ulex australis*). Figure 3 shows the sequence of analyses performed, specifying the phenomena analyzed and their corresponding interpretation.

#### 2.3.1 Community analyses

The main gradients of vegetation composition were described with a NMS (Nonmetric Multidimensional Scaling) ordination of shrub cover data, with the function metaMDS of R Package “vegan” (Oksanen et al., 2013). The dissimilarity between plots was measured with the Bray‐Curtis distance, and the goodness‐of‐fit of the NMS ordination was assessed through the percentage of variance represented by each consecutive axis (see McCune & Grace, 2002 for details). GAM analyses were performed using the “mgcv” R package (Wood, 2006) to characterize and compare the relationships of community composition gradients identified from NMS analyses with the abundance of the *S. genistoides* and *U. australis* along the four datasets (from the three study regions and the whole extent) and describe the variations in the species and community co‐occurrence throughout the studied regions.

#### 2.3.2 Community co‐occurrence

We calculated checkerboard scores (C‐score, Stone & Roberts, 1990) to assess whether there was any spatial association between the dominant species of both communities, i.e. *S. genistoides* and *U. australis*, and also if their patterns of co‐occurrence were consistent throughout the three regions. To do this, we used the “*C.score*” function of the “bipartite” R software package (Dormann et al., 2008), and determined significance thresholds for these C‐scores under a null model by performing 999 community simulations preserving site and species' frequencies (Barberán et al., 2012; Götzenberger et al., 2016) through the “*oecosimu*” function of “vegan” R package (Oksanen et al., 2013). Low C‐score values indicate high randomness in species distributions whilst high values suggest non‐random species distributions. Data normalization implies that this index ranges between 0 (no checkerboards, that is, the presence of one species is independent of the presence or absence of the other species) and 1 (only checkerboards, i.e. the two species never coexist). In the presence of competitive exclusion or any other kind of repulsion (sensu Keil et al., 2021), we will expect values closer to one, highlighting the importance of antagonistic biotic interactions throughout the successional dynamics reported between *S. genistoides* and *U. australis* communities. In contrast, values closer to zero imply that biotic interactions are not driving the prevalence of either community. C‐sore analyses were performed using the cover data on *S. genistoides* and *U. australis* cover in all sampled sites and each one of the three regions separately. All analyses were performed using R statistical software V.3.6.3 (R Core Team, 2022) and the R Studio V 0.98.1103 interface.

#### 2.3.3 Determinants of community variations

We analyzed how shrub community variations are affected by: (i) soil organic matter, a local factor (explained above); (ii) mesoscale environmental gradients in aridity and a broad set of climatic, topographic, and lithological variables; (iii) the individualistic responses of *S. genistoides* and *U. australis* to these mesoscale gradients, measured as habitat suitability scores from Species Distribution Models (SDMs).

Altitude and climatic variables (*n* = 34, see Table S1—Appendix S2) were extracted from WorldClim (Fick & Hijmans, 2017), and lithological variables from EDIT GeoPlatform (Sastre et al., 2009). We calculated aridity as the aridity index (Ia) used by Rivas‐Martínez (1987) to define Mediterranean vegetation zones: Ia = 1/(P/T + 10) × 100, where P is the mean annual precipitation and T means annual temperature. Lithological variables were extracted from the lithological maps of Portugal (APA, 1992) and Spain (IGME, 1994), and distance to coast was calculated using ArcGIS 10.2 (ESRI, 2019). Multicollinearity among environmental variables was handled by dropping collinear covariates when correlated at Spearman r > |0.8| (Dormann et al., 2012) and opting for the variables that allow easier and meaningful interpretations (Zuur et al., 2010).

We modeled the realized environmental niche of *S. genistoides* and *U. australis* using these environmental variables as predictors and considering their entire distribution in the Iberian Peninsula. We obtained occurrence records for each species at a 10 km × 10 km resolution from the main herbaria holding Iberian Mediterranean collections—COI (63 specimens), LISI (62), LISU (50), MA (44), MACB (5), MAF (19), and SEV (4); herbarium abbreviations according to the Index Herbariourum (https://sweetgum.nybg.org/science/ih/)—Flora‐on platform (Chozas, Carapeto, et al., 2015; Chozas, Porto, et al., 2015), Anthos database (http://www.anthos.es/), and our own data gathered from field work. We used ENFA (Ecological Niche Factor Analysis; Hirzel, Hausser, Chessel, et al., 2002), a presence‐only SDM technique, to model the realized scenopoetic niche of the selected species—that is, the quantitative set of abiotic conditions of the sites where the species occurs in the studied region (Hortal, Lobo, et al., 2012; Hutchinson, 1978; Soberón, 2007). ENFA is particularly adequate to model species' realized niches. Briefly, it is an ordination technique that identifies the set of orthogonal factors that best characterize the response of the species to the environmental conditions present in the region. This analysis identifies a Marginality Factor—that accounts for the direction of maximum difference between the conditions occupied by the species and all available conditions in the study area, as well as one or several Specialization Factors—that quantify the ecological variance of the species in relation to other progressively less important environmental gradients. The responses to these factors can then be used to project the realized response of the species back into the geographical space. Further, ENFA allows for calculating the overall Marginality and Specialization of the species, that characterize how far its response is from the predominant conditions in the region, and how much it is affected by minor environmental and habitat gradients (Hirzel, Hausser, & Perrin, 2002).

ENFA allows quantifying the contribution of each variable to the marginality and specialization factors, thereby providing a description of the realized response of the species to each predictor. Therefore, we first performed a preliminary ENFA to select the set of uncorrelated variables (in terms of marginality and specialization) that best explain the distribution of the species, in order to avoid problems of multicollinearity. Subsequently, we used those selected variables to perform a definitive ENFA to model the realized environmental niche of each species and estimate the importance of each predictor and the global marginality coefficients of the different species. Finally, species' potential distributions were calculated based on the variables selected in the first step by means of Mahalanobis distance (MD; Farber & Kadmon, 2003). This presence‐only species distribution modeling algorithm generates an elliptic climate envelope of the response of the species in the multidimensional space defined by the predictor variables, around the optimum conditions. MD allows projecting a habitat suitability map by means of obtaining the distance to such optimum in the environmental space (Clark et al., 1993). ENFA and MD were fit using the “adehabitat” R Package (Calenge, 2006). Differences in the habitat suitability of each region for *S. genistoides* and *U. australis* were calculated by performing paired Wilcoxon tests. Bonferroni correction was used for multiple testing. To evaluate ENFA models we used the Boyce index (Bi; Boyce et al., 2002). Bi ranges continuously from −1 to 1, where positive values indicate consistent predictions and 0 means a random model (Hirzel et al., 2006). We calculated Bi with “ecospat” package in R (Di Cola et al., 2017).

Further, to identify the geographical variations in the effect and importance of local and regional drivers of community assembly we examined the relationships between the NMS ordination and the set of explanatory variables resulting from previous analyses through vector fitting, using the function “*envfit*” of R package vegan.

## 3 RESULTS

**FIGURE 4:**
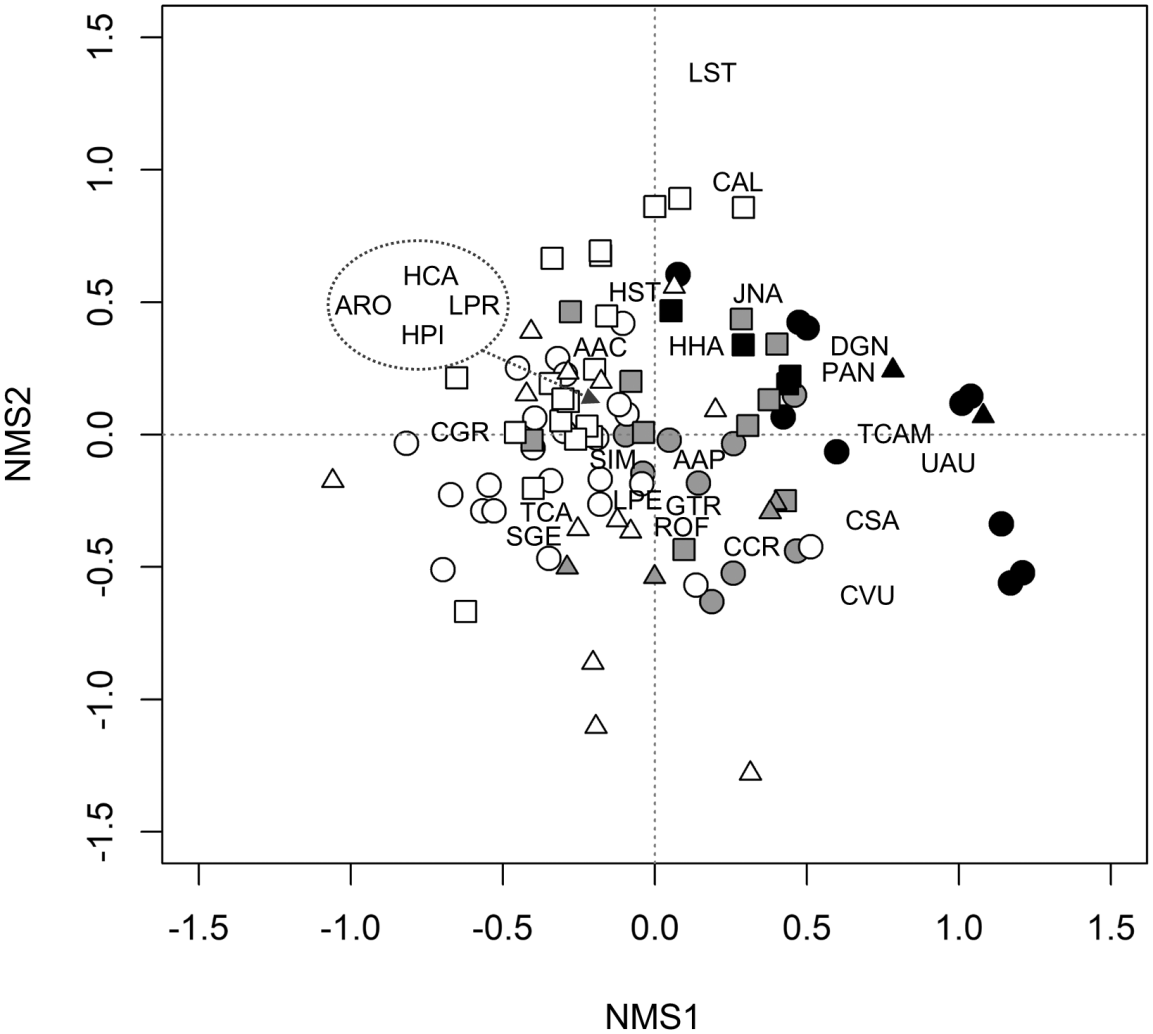
First two axes (NMS1 and NMS2) of the 3‐dimensional non‐metric multidimensional scaling ordination of all study sites based on shrub cover. Final stress = 0.19. Empty symbols represent study sites with *Stauracanthus genistoides* (SGE) but without *Ulex australis* (UAU), black symbols do so for study sites with *U. australis* but without *S. genistoides*, and gray symbols are study sites with both species. Circles correspond to Setúbal Peninsula sites, squares to Comporta sites, and triangles to sites located in the South/South‐Western region. To guarantee the readability of the figure, only the 25 shrubs occurring in more than 5% of the plots (i.e. with more than five occurrences) are shown. Species codes are listed in Table S2 – Appendix S3.

The non‐metric multidimensional scaling identified three axes describing variations in shrub cover with a final stress value of 0.18. Shrub cover values of the 47 shrub species registered (Table S2 – Appendix S3) were square rooted to reduce the influence of large values. The first and second axes (herein NMS1 and NMS2) described the main gradients in species composition and account for most of the variance of the ordination (44.44% out of 65.08%) (Figure 4). In particular, NMS1 identified the gradient from sites dominated by *Stauracanthus genistoides* (negative values) or *Ulex australis* (positive values).

**FIGURE 5:**
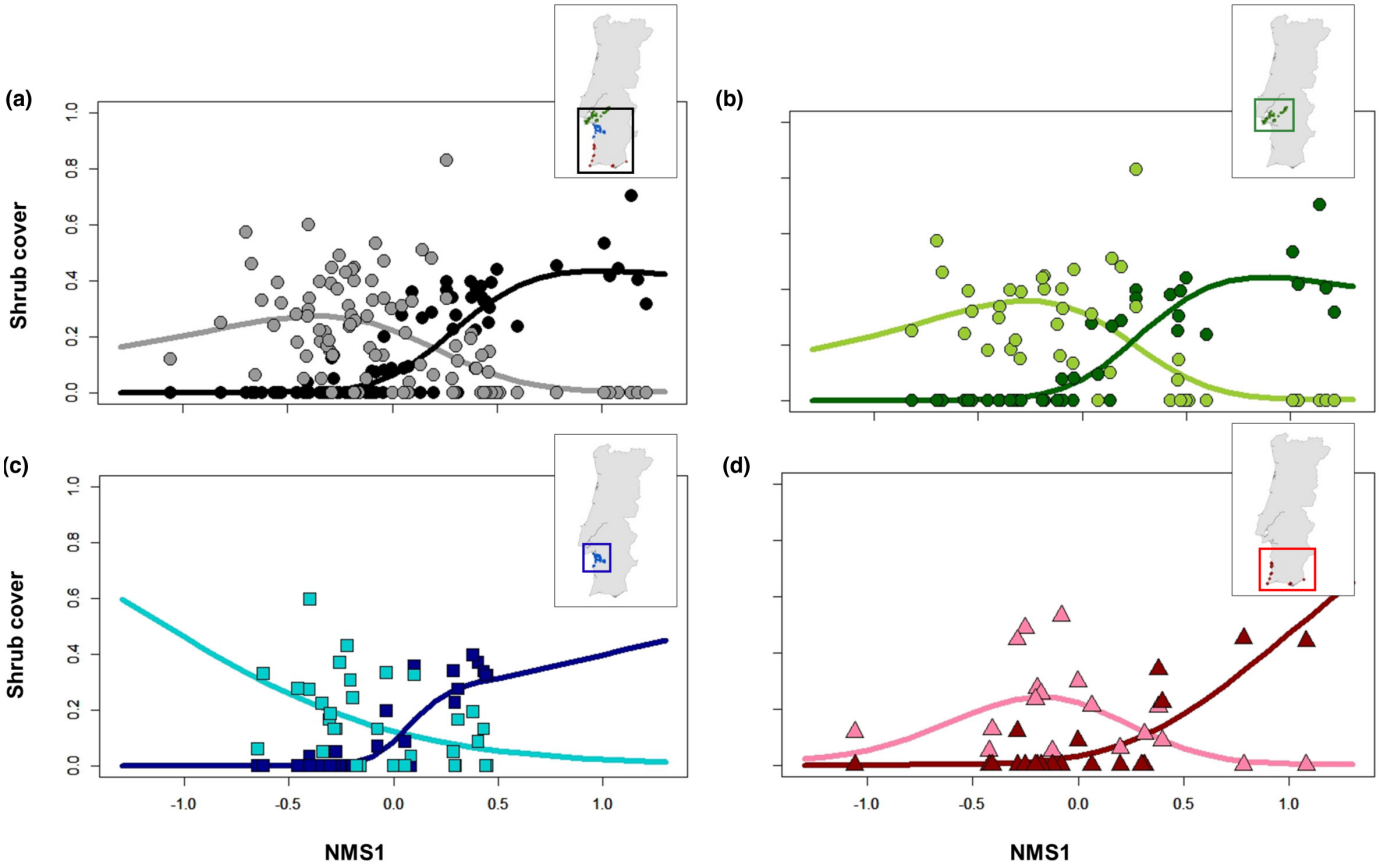
Relationships between the cover of *Stauracanthus genistoides* (pale colored lines) and *Ulex australis* (dark colored lines), and the first axis of the ordination based on shrub cover representing the gradient between the communities dominated by these two species (NMS1) in: (a) all sites (black/gray), (b) Setúbal Peninsula (green circles), (c) Comporta region (blue squares), and (d) South/South‐Western region (SSW) (pink and red triangles). The main trends of the relationships were identified by Generalized Additive Models of the binomial family. See Table S3—Appendix S4 for the Deviance explained, p, k, and n of GAM models.

GAMs performed to characterize and visually compare the relationships between NMS1 and the cover of *S. genistoides* and *U. australis* for each region, identified a succession‐like pattern of replacement of *S. genistoides* by *U. australis*, and their respective communities, that is consistent both along the compositional gradients and across all regions (Figure 5).

C‐scores identified significant spatial segregation between *S. genistoides* and *U. australis* throughout the whole study area (C‐score = 0.57, *p* < .01). However, at a smaller scale, this relationship only held up in the Setúbal region (C‐score = 0.87, *p* < .01), while in Comporta and SSW regions C‐score values did not differ significantly from those generated by the null models.

**FIGURE 6:**
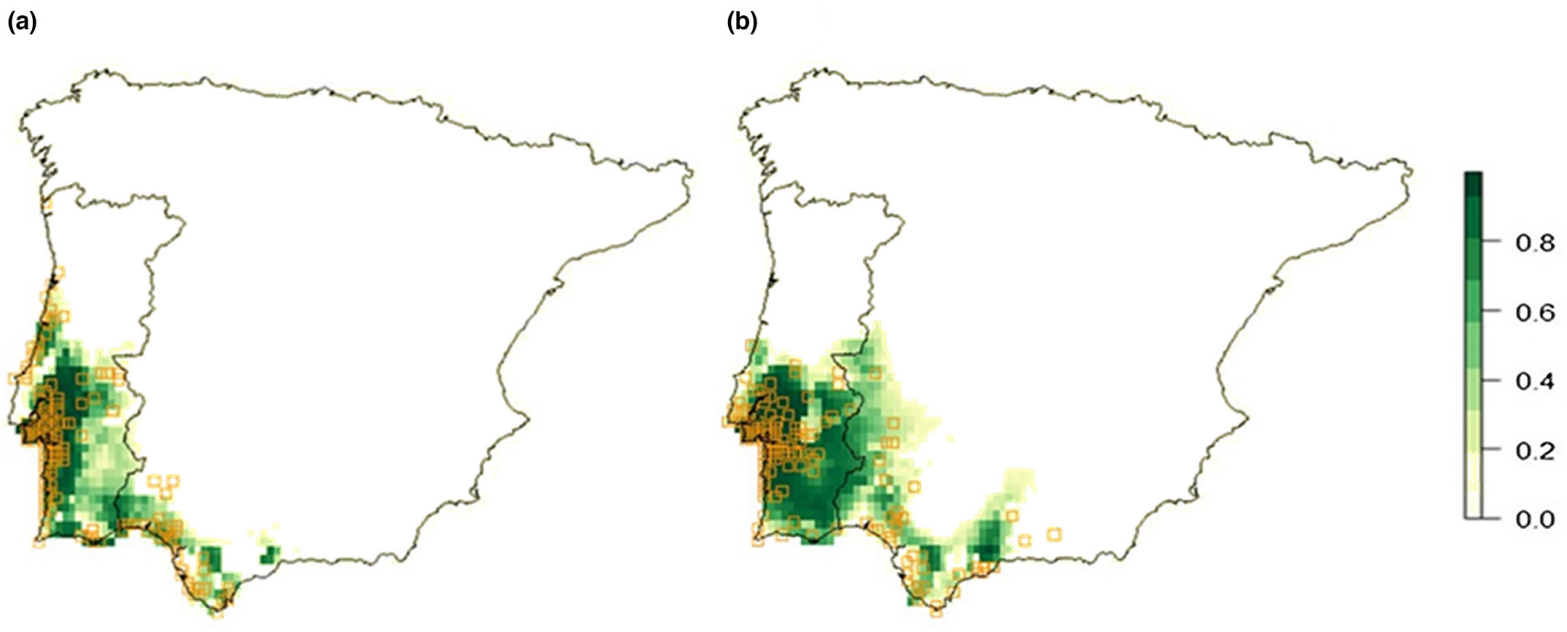
Maps of the environmental suitability in the Iberian Peninsula for the dominant species of the two community types identified in this study (a—*Stauracanthus genistoides*; b—*Ulex australis*), derived through Ecological Niche Factor Analysis (ENFA) analyses. Empty yellow squares indicate the known occurrences of both species that were used to calibrate and validate these analyses. Increasing degrees of green indicate higher levels of habitat suitability.

Eleven uncorrelated environmental variables were evaluated as predictors of the distribution of *S. genistoides* and *U. australis* in the preliminary ENFA. Six variables held significant contributions to marginality and specialization factors (Table 1) and were used to model the realized scenopoetic niches of these two species. Mean annual temperature, isothermality, and temperature seasonality accounted for most of the marginality of the two species. Both species occur in localities with higher mean temperature and isothermality and lower seasonality than the average conditions in the Iberian Peninsula. Further, mean monthly radiation and mean temperature of the wettest quarter were the most influential for the specialization factors of *S. genistoides* and *U. australis*, respectively. This indicates a more restricted range of ecological variance in relation to these predictors within the study area. Habitat suitability maps of *S. genistoides* and *U. australis* were then calculated with the six significant predictors (Figure 6). The model calibrated for *S. genistoides* achieved better performance (Bi = 0.747) than the one for *U. australis* (Bi = 0.247), probably because *U. australis* has a wider climatic niche (marginality score = 4.09) than *S. genistoides* (marginality score = 5.29). Although both species occur only on sandy soils, ENFA analyses did not select any edaphic variables as relevant for the models. Thus, *U. australis* suitable habitat extension is larger (Figure 6). Results of Paired Wilcoxon tests showed significant differences between the habitat suitability of *S. genistoides* and *U. australis* across the three regions. SSW region presents the lowest levels of habitat suitability of the studied area (average of 0.60 for *S. genistoides* and 0.52 for *U. australis*, compared to an average of 0.98 and 0.99 and 0.93 and 0.98 in Setúbal Peninsula and Comporta, respectively).

**TABLE 1:** Ecological Niche Factor Analysis (ENFA) results of *Stauracanthus genistoides* (SGE) and *Ulex australis* (UAU) with the six more informative variables (after variable selection).

	Scores SGE		Scores UAU	
	Mar	Spe1	Mar	Spe1
Annual mean temperature	0.51	0.10	0.56	0.06
Annual precipitation	0.02	−0.12	−0.03	−0.23
Isothermality	0.49	0.00	0.52	−0.10
Average monthly radiation	0.07	−0.98	0.05	0.25
Mean temperature of wettest quarter	0.24	−0.08	0.21	−0.88
Temperature seasonality	−0.66	−0.06	−0.61	−0.31
Marginality	5.29		4.09	

Abbreviations: Mar, marginality axis; Spe1, first specialization axis.

**FIGURE 7:**
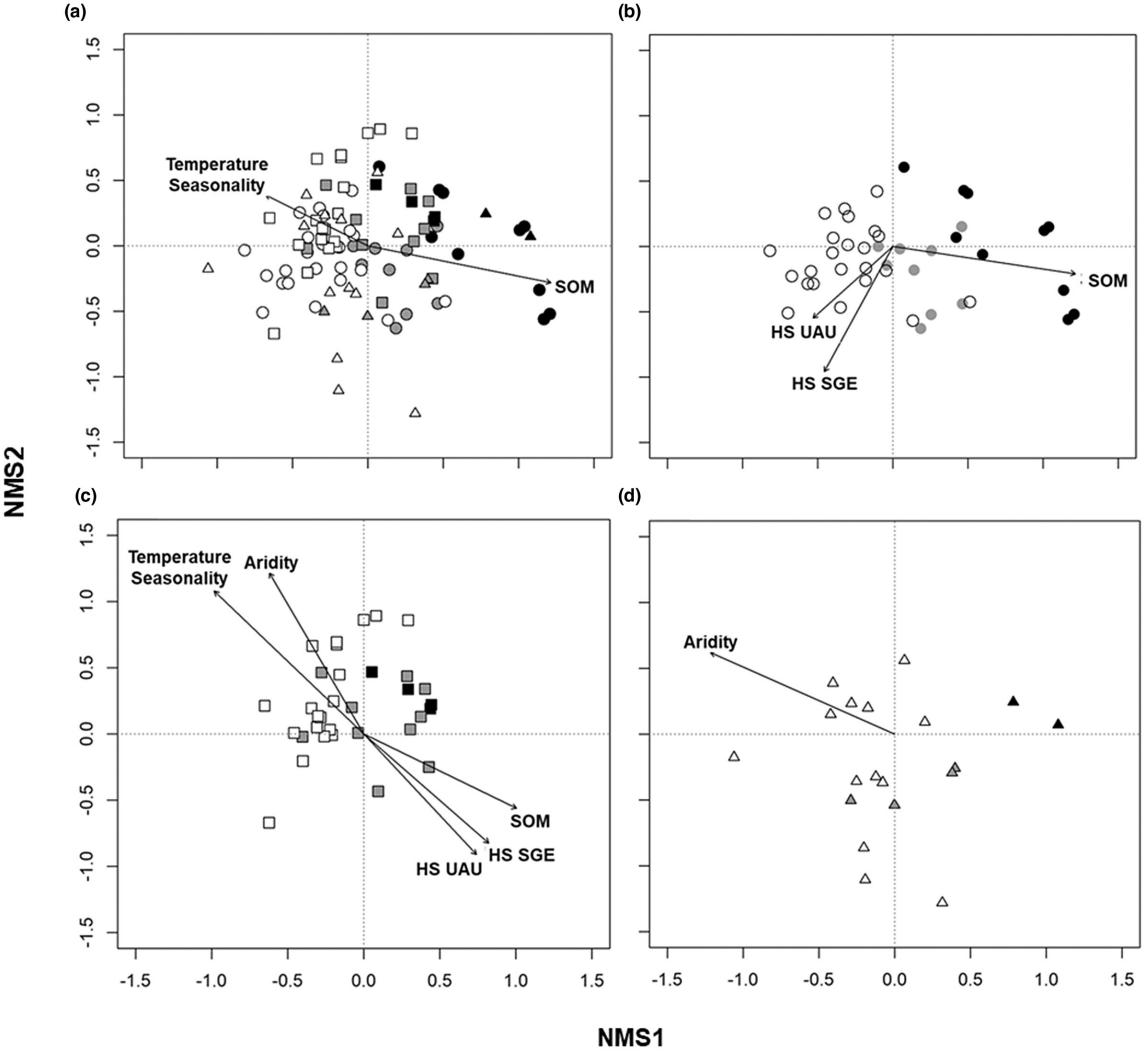
First two axes (NMS1 and NMS2) of the 3‐dimensional non‐metric multidimensional scaling ordination of study sites based on shrub cover. Vectors represent significant correlations between environmental variables and the NMS ordination of (a) all study sites and for each region individually, namely: (b) Setúbal, (c) Comporta, and (d) South/South‐Western region (SSW). Empty symbols represent study sites with *Stauracanthus genistoides* (SGE) but without *Ulex australis* (UAU), black symbols represent study sites with *U. australis* but without *S. genistoides*, and gray symbols are study sites with both species. Circles correspond to Setúbal Peninsula sites, squares to Comporta sites, and triangles to sites located in the South/South‐Western region.

Spearman correlations between the complete set of potential predictors for the NMS ordination were used to eliminate collinear covariates, so the final selected variables were: soil organic matter (SOM), aridity, mean diurnal temperature range, temperature seasonality, and habitat suitability (HS) of *S. genistoides* and *U. australis*. Then, two predictors were selected to explain the NMS ordination of all the study sites through vector fitting: SOM (*R* ^2^ = 0.27, *p* < .001) and Temperature Seasonality (*R* ^2^ = 0.10, *p* < .01) (Figure 7a). SOM content (*R* ^2^ = 0.50, *p* < .001) and the HS of both *S. genistoides* and *U. australis* (*R* ^2^ = 0.37, *p* < .001 and *R* ^2^ = 0.19, *p* < .01, respectively) also described the gradient between the two communities in the Setúbal region, according to vector fitting (Figure 7b). However, in the Comporta region, temperature seasonality (*R* ^2^ = 0.57, *p* < .001), aridity (*R* ^2^ = 0.50, *p* < .001), HS of *S. genistoides* and *U. australis* (*R* ^2^ = 0.36, *p* < .05 and *R* ^2^ = 0.37, *p* < .001, respectively) and SOM (*R* ^2^ = 0.35%, *p* < .01), explained the gradient (Figure 7c). In contrast, aridity is the sole main driver explaining the gradient in the South/South‐western (SSW) region (*R* ^2^ = 0.51, *p* < .01) (Figure 7d).

## 4 DISCUSSION

Overall, our results show that the relative importance of local and regional factors causing community‐level responses varies according to the suitability of the environmental conditions for the dominant species in the community. Although soil organic matter (SOM) and climate variables are key determinants of variations in the composition of the Iberian xerophytic shrub communities of inland dunes, their importance varies along the studied gradient. Indeed, SOM is the main driver of species turnover between *S. genistoides* and *U. australis* communities throughout both the whole study area and Setúbal region, partially consistent with the preponderance of local processes as determinants of the composition of these communities (see also Chozas, Correia, et al., 2015). This contrasts with the Comporta and South/South‐Western (SSW) regions, where most of the variation between both types of communities is explained by climate: temperature seasonality and aridity in the former and only aridity in the latter. Therefore, in this study system, the substitution of the communities dominated by *S. genistoides* by those dominated by *U. australis* is remarkably consistent throughout the whole area shared by both species, although the variables that act as main drivers of the change between both types of communities shift in importance throughout a clear N–S gradient. That is, while the patterns of compositional variation are homogeneous along space, the importance of the factor(s) driving it changes from one region to another. This suggests that similar successional processes can be triggered by different factors, depending on the main constraints affecting the dominant species of the community in each region. This adds a new perspective to the species‐specific effects of environment on community dynamics.

Understanding how different community‐ and niche‐based processes acting on a similar species pool in nearby regions lead to very similar patterns is not straightforward. Our results indicate that other mechanisms not taken into consideration in this study may be regulating the effects (and thus the relevance) of the main factors acting in the transition between different community stages. Although the gradient between *S. genistoides* and *U. australis* communities responds largely to the amount of organic matter in the soil for the whole extent of the studied area, it does so with different intensities. While in Setúbal Peninsula the gradient between both communities is highly correlated with this factor, its effect is not significant in SSW, and climate factors are more relevant as determinants of this sequence in Comporta.

The Setúbal Peninsula's complex edaphic matrix is distinguished by older, more consolidated, and SOM‐richer sandy soils than those of Comporta. These edaphic features perfectly match the requirements of *U. australis* (Chozas, Correia, et al., 2015). Interestingly, C‐score values indicate that the occurrence of *S. genistoides* is highly conditioned by the presence of *U. australis* in Setúbal Peninsula, identifying a significant spatial segregation between both communities. However, the C‐scores indicate a random pattern of co‐occurrence of both species in Comporta, probably reflecting the pattern described by Neto et al. (2007) for the sandy soils on the Tagus river basin, where Setúbal Peninsula region is located. These authors proposed that human activities, mainly plowing, reduce sand aggregation and induce pioneer psammophilous species to colonize disturbed sandy soils. Consequently, because of the sand remobilization, these soils present lower levels of soil water retention and soil organic matter (Huntington, 2006), favoring the occurrence of *S. genistoides* communities and preventing the establishment of *U. australis* communities. In contrast, Neto et al. (2004) suggested that the presence of a sub superficial layer of sand cemented by iron oxides in Comporta region in the past acted as a barrier to water, improving humid edaphic conditions in sandy soils. This would have facilitated the preservation of a relict and more hygrophytic community in Comporta, which is characterized by the co‐dominance of *U. australis* and heaths (mainly *Erica umbellata* and *E. scoparia*). Similar to Setúbal Peninsula, historical and recent agricultural and forestry practices destroyed most of the cemented layer, and therefore the hygrophilous species (mainly heaths) have already disappeared from these communities. Nevertheless, the biological legacy generated by this layer (sensu Franklin et al., 2000; see also MacMahon, 1980), and the presence of compensatory processes (see resistance and resilience theory by Connell & Ghedini, 2015), may provide some mitigating effect to the resultant disturbance. This may have preserved the occurrence of *U. australis* communities on the sandy soils of Comporta but following a random and/or spatially unstructured pattern depending on historical events. Indeed, several Portuguese national programmes, such as the Wheat Campaign (1929), the Irrigation Plan (1935), and the Afforestation Plan (1938), have had a significant influence on Portuguese agricultural and forestry landscapes (Sucena‐Paiva et al., 2022).

In conclusion, we believe that the results of the C‐score analyses point to successional mechanisms in the Setúbal region, while local contingencies associated with historical processes affecting the surface layers of inland dunes could have defined community dynamics in both Setúbal and, to a higher degree, Comporta regions. This links past human disturbances and previous configurations of the superficial soil layer to the current structure of these communities, highlighting that landscapes with similar environmental conditions but subject to different historical processes can result in different community dynamics (Dark, 1990).

In clear contrast, the studied succession seems to be determined by different processes in the South/South‐Western region, associated with large‐scale climatic gradients rather than local variations in species coexistence and soil organic matter. Here, the two dominant species present the lowest levels of habitat suitability found in the whole studied area. Since SSW communities are far from their climatic optimum, climatic conditions constitute a main constraint and therefore their distribution is more dependent on them. Consequently, *S. genistoides* prevails in areas with higher aridity values, while *U. australis* dominates in milder climate areas. Here, we must note that following Pardo et al. (2008) our ENFA analyses were based on data from *S. genistoides* aggr, including *S. genistoides* and *S. spectabilis*, whose taxonomic status is, at least, uncertain. This provided a conservative indicator of the climatic niche‐based suitability of the monophyletic clade of *S. genistoides* sensu lato, under the sake of potentially missing the small differences between the niche‐based responses of *S. spectabilis* and *S. genistoides* sensu stricto. Given the high similarity between their responses, it is unlikely that this has any effect on the generality of our results, although some small differences in local responses may appear especially if data from more extensive surveys were available, allowing to perform more detailed analyses on the responses of the populations from these two terminal branches of the *Stauracanthus* tree.

Several species interactions are also consistently shaping up the composition dynamics of the studied xerophytic shrub communities. A previous study conducted only in the northern distribution of these communities (Chozas, Carapeto, et al., 2015) attributed the initial stages of the gradient between *S. genistoides* and *U. australis* communities to a process of facilitation, through the accumulation of organic matter in the soil. This would explain the predictive power of this variable mainly in the Setúbal Peninsula. This contrasts with the lack of effects of soil organic matter on the replacement of *S. genistoides* by *U. australis* found in the SSW region, where both species are far from their realized climatic optimum. Here, it is likely that competition for occupying the small pockets of sand of heterogeneous origin, determines replacement between the two types of communities. In fact, competition is also part of the process of community replacement described by Chozas, Correia, et al. (2015); after establishing, the species from the community dominated by *U. australis* outcompete those present in the early stages dominated by *S. genistoides*. Consequently, we believe that in the SSW region, competitive exclusion plays a major role in maintaining the geographical consistency of the sequence between communities throughout largely differentiated environmental conditions. In areas that are optimal for both species, *S. genistoides* is a pioneer in colonizing sands with no or poor soil development, which allows the more edaphically exigent species of the *U. australis* communities to establish later on. The species that form these latter communities will prevail in all patches of sand available in the areas with harsher climatic conditions. Whereas species from *S. genistoides* communities will dominate only when conditions are so severe that *U. australis* communities cannot thrive.

To summarize, we show a remarkably consistent sequence of community compositional changes throughout the heterogeneous soil and climate conditions of the inland sand dune habitats of the south of Portugal. Rather than finding consistency in the determinants of such successional sequence, our results evidence that both communities and their dominant species respond in a similar way to different environmental gradients and, probably, landscape disturbance history in different regions. These results highlight the importance of integrating niche‐based species responses to large‐scale environmental gradients in our understanding of local community dynamics. According to this, we can conclude that the preponderance of local species interactions and microenvironmental conditions in determining assembly and successional processes should be at its utmost in the regions with conditions near to the optima of the dominant species, progressively diminishing in favor of climatic gradients in the regions placed near the limits of their climatic tolerances.

## AUTHOR CONTRIBUTIONS

**Sergio Chozas:** Conceptualization (equal); formal analysis (lead); investigation (lead); writing – original draft (lead); writing – review and editing (lead). **Rosa M. Chefaoui:** Conceptualization (equal); formal analysis (equal); writing – review and editing (supporting). **Ana M.C. Santos:** Conceptualization (equal); writing – review and editing (supporting). **Otilia Correia:** Conceptualization (equal); supervision (equal); writing – review and editing (equal). **Joaquín Hortal:** Conceptualization (equal); funding acquisition (lead); supervision (equal); writing – original draft (lead); writing – review and editing (lead).

### OPEN RESEARCH BADGES

This article has earned Open Data and Open Materials badges. Data and materials are available at Appendix S5.

## Supporting information


Appendix S1.

Appendix S5:

## Data Availability

Primary data are presented as Appendix S5 Primary data.
