# Supplementary material for: Geographical shifts in the successional dynamics of inland dune shrub communities
Source: Ecol Evol. 2023 Feb 16;13(2):e9828. doi: 10.1002/ece3.9828 (PMC9935296; doi:10.1002/ece3.9828)
Supplement: Supplementary file 1 — Appendix S1. [file ECE3-13-e9828-s002.docx]

**SUPPORTING INFORMATION**

**Geographical shifts in the successional dynamics of inland dune shrub communities**

Sergio Chozas, Rosa M. Chefaoui, Otília Correia,
Ana M. C. Santos and Joaquín Hortal

Appendix S1. Dominant land use in the study area


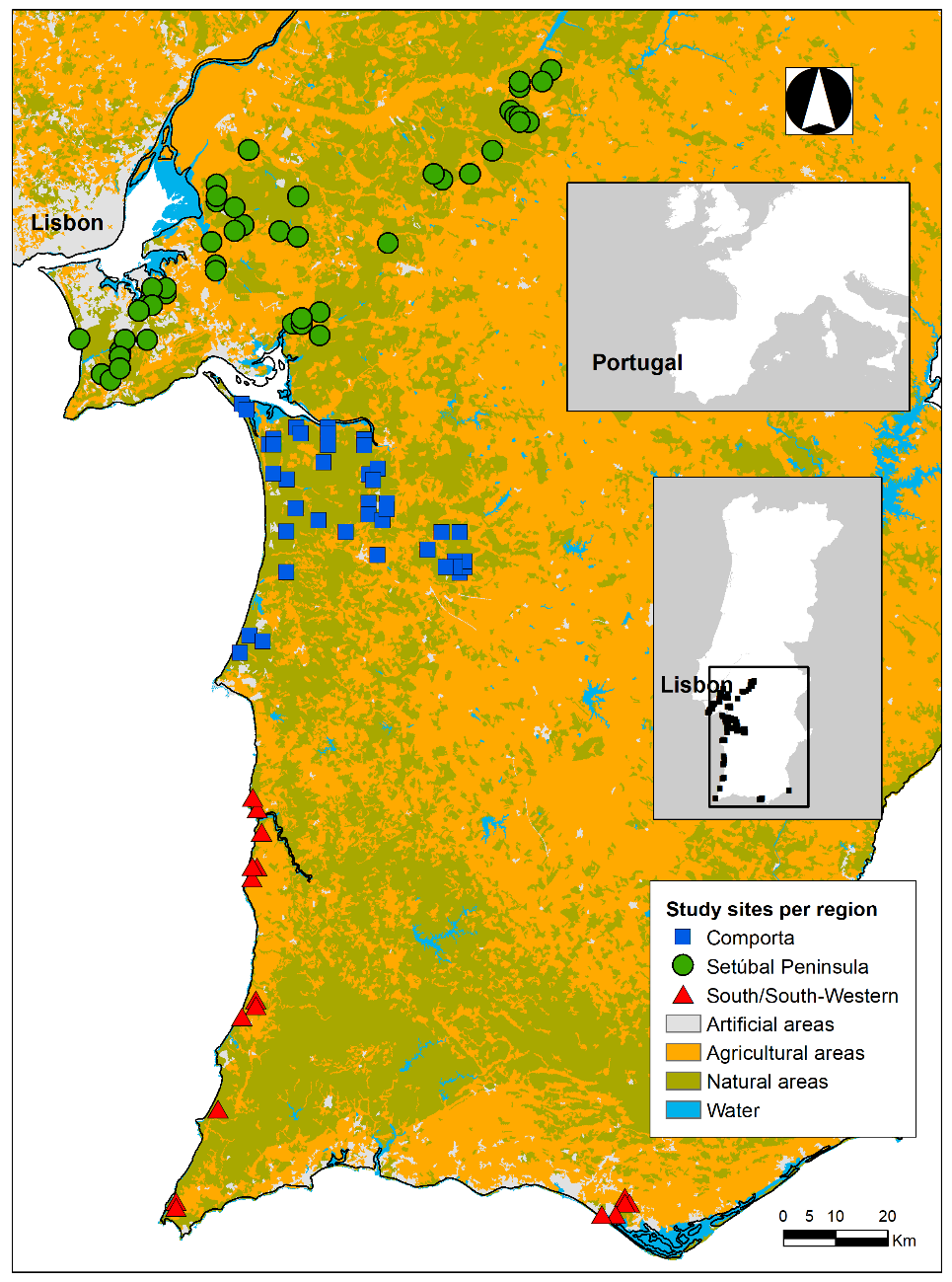


**Figure S1** Study sites and dominant land use in the study area (Corine, 2018). Sites were divided in three regions – Setúbal Peninsula (green circles), Comporta (blue squares), and South/South-Western region (SSW) (red triangles).

Appendix S2. Climatic, topographic and lithological variables used in community analyses.

**Table S1** Climatic, topographic and lithological variables initially available. All variables were extracted from Worldclim (Hijmans et al. 2005), except 1) (obtained from Sastre et al. 2009), 2) (calculated using ArcGis 10.2; ESRI 2011) and 3) (APA 1992; IGME 1994).

| **Climatic and topographic variables** | |
| --- | --- |
| Annual Mean Temperature (ºC) | Min Temperature of Coldest Month (ºC) |
| Actual evapotranspiration (mm)^1)^ | Precipitation of Coldest Quarter (mm) |
| Annual Precipitation (mm) | Precipitation of Driest Month (mm) |
| Aridity Index^1)^ | Precipitation of Driest Quarter |
| Average Monthly Maximum Temperature | Precipitation of Warmest Quarter (mm) |
| Average Monthly Mean Temperature (ºC) | Precipitation of Wettest Month (mm) |
| Average Monthly Minimum Temperature (ºC) | Precipitation of Wettest Quarter (mm) |
| Average Monthly Precipitation (mm) | Precipitation Seasonality (mm) |
| Average Monthly Radiation^1)^ | Real evapotranspiration (mm)^1)^ |
| Hydric balance (mm)^1)^ | Temperature Annual Range (ºC) |
| Isothermality | Temperature Seasonality |
| Max Temperature of Warmest Month (ºC) | Elevation Range (m) |
| Mean Diurnal Range (ºC) | Distance to the coast (km)^2)^ |
| Mean Temperature of Coldest Quarter (ºC) | Distance to Pyrenees (km)^1)^ |
| Mean Temperature of Driest Quarter (ºC) | Maximum Elevation (m) |
| Mean Temperature of Warmest Quarter (ºC) | Mean Elevation (m) |
| Mean Temperature of Wettest Quarter (ºC) | Minimum Elevation (m) |
| **Lithological variables^3)^** | |
| Holocene and Pleistocene sedimentary substrates | Miocene and Pliocene sedimentary substrates |
| Metamorphic and sedimentary substrates (excluding prior classes) | Plutonic substrates |
| Volcanic substrates |  |

APA 1992. Atlas do ambiente. Agência Portuguesa do Ambiente. Lisbon.

ESRI 2011. ArcGIS Desktop: Release 10. Redlands, CA: Environmental Systems Research Institute.

Hijmans, R. J. et al. 2005. Very high resolution interpolated climate surfaces for global land areas. - Int. J. Climatol. 25: 1965–1978.

IGME 1994. Mapa geológico de España. Instituto Geológico y Minero de España. Madrid.

Sastre, P. et al. 2009. A Geoplatform for improving accessibility to environmental cartography. - J. Biogeogr. 36: 568–568.

Appendix S3. Species names of the surveyed shrubs.

**Table S2** Species names of the surveyed shrubs. Codes, only for the 25 more abundant species, were used in Figure 3.

| **Shrub species** | **Code** | **Shrub species** | **Code** |
| --- | --- | --- | --- |
| *Armeria rouyana* Daveau | ARO | *Lavandula stoechas* L. subsp. *stoechas* | LST |
| *Asparagus acutifolius* L. | AAC | *Lavandula pedunculata* (Mill.) Cav.  subsp. *pedunculata* | LPE |
| *Asparagus aphyllus* L. | AAP | *Lithodora prostrata* (Loisel.) Griseb.  subsp. *lusitanica* (Samp.) Valdés | LPR |
| *Calluna vulgaris* (L.) Hull | CVU | *Ononis ramosissima* Desf*.* |  |
| *Cistus crispus* L. | CCR | *Osyris lanceolata* Hochst. & Steud. |  |
| *Cistus ladanifer* L. |  | *Phagnalon rupestre* (L.) DC. |  |
| *Cistus libanotis* L. |  | *Phillyrea angustifolia* L. | PAN |
| *Cistus monspeliensis* L. |  | *Pistacia lentiscus* L. |  |
| *Cistus psilosepalus* Sweet |  | *Pterospartum tridentatum* (L.) Willk. |  |
| *Cistus salviifolius* L. | CSA | *Quercus coccifera* L. |  |
| *Corema album (L.)* D. Don | CAL | *Quercus lusitanica* Lam. |  |
| *Cytisus grandiflorus* (Brot.) DC. subsp*. cabezudoi* Talavera | CGR | *Retama monosperma* (L.) Boiss. |  |
| *Daphne gnidium* L. | DGN | *Rosmarinus officinalis* L. | ROF |
| *Dianthus broteri* Boiss. & Reut. |  | *Santolina impressa* Hoffmanns. & Link | SIM |
| *Erica scoparia* L. |  | *Stauracanthus boivinii* (Webb) Samp. |  |
| *Erica umbellata* L*.* |  | *Stauracanthus genistoides* (Brot.) Samp. | SGE |
| *Genista triacanthos* Brot*.* | GTR | *Teucrium vicentinum* Rouy |  |
| *Halimium calycinum* (L.) K.Koch | HCA | *Thymus albicans* Hoffmanns. & Link |  |
| *Halimium halimifolium* (L.) Willk. | HHA | *Thymus camphoratus* Hoffmanns. & Link | TCAM |
| *Helianthemum apenninum* subsp. *stoechadifolium* (Brot.) Samp. |  | *Thymus capitellatus* Hoffmanns. & Link | TCA |
| *Helichrysum italicum* (Roth) G. Don subsp. *picardi* (Boiss. & Reut.) Franco | HPI | *Thymus carnosus* Boiss. |  |
| *Helichrysum stoechas* (L.) Moench subsp. *stoechas* |  | *Ulex argenteus* Welw. ex Webb |  |
| *Juniperus navicularis* Gand. | JNA | *Ulex australis* Clemente | UAU |
| *Juniperus turbinata* subsp*. turbinata* Guss*.* |  |  |  |

Appendix S4. GAM analyses results characterising the community composition gradients with the abundance of the *S. genistoides* and *U. australis*

**Table S3** Deviance explained, p, k and n of GAM models between S. genistoides (SGE) and *U. australis* (UAU) cover and the first community axis (NMS1) values in each region.

|  |  | **Deviance explained (%)** | **p** | **k** | **n** |
| --- | --- | --- | --- | --- | --- |
| **All sites** | **SGE** | 26.8 | <0.001 | 1.989 | 95 |
|  | **UAU** | 69.4 | <0.001 | 1.995 | 95 |
| **Setúbal** | **SGE** | 48 | <0.001 | 1.998 | 42 |
|  | **UAU** | 71 | <0.001 | 1.991 | 42 |
| **Comporta** | **SGE** | 19.1 | <0.001 | 1.001 | 33 |
|  | **UAU** | 77.5 | <0.001 | 1.999 | 33 |
| **SSW** | **SGE** | 28.2 | <0.001 | 1.98 | 20 |
|  | **UAU** | 66.1 | <0.001 | 1.911 | 20 |
